# Differences in the Use of Primary Care Services Between Spanish National and Immigrant Patients

**DOI:** 10.1007/s10903-012-9647-x

**Published:** 2012-05-22

**Authors:** L. A. Gimeno-Feliu, R. Magallón-Botaya, R. M. Macipe-Costa, L. Luzón-Oliver, J. L. Cañada-Millan, M. Lasheras-Barrio

**Affiliations:** 1 Aragon Health Sciences Institute (IACS), Zaragoza, Spain; 2 Aragonese Primary Care Research Group-Research Network on Preventative Activities an Health Promotion (redIAPP), Zaragoza, Spain; 3 Departament of Medicine, Zaragoza University, Zaragoza, Spain; 4 San Pablo Health Centre, SALUD (Aragon Health Service), Aguadores St 7, 50003 Zaragoza, Spain; 5 Arrabal Health Centre, SALUD, Zaragoza, Spain; 6 Fuentes de Ebro Health Centre, SALUD, Zaragoza, Spain; 7 Canal Imperial Health Centre, SALUD, Zaragoza, Spain; 8 Hellin Health Centre, SESCAM (Castilla-La Mancha Health Service), Albacete, Spain

**Keywords:** Emigrants and immigrants, Health services/utilization, Primary health care, Healthcare disparities/statistics and numerical data, Spain

## Abstract

Knowing what real use is made of health services by immigrant population is of great interest. The objectives are to analyze the use of primary care services by immigrants compared to Spanish nationals and to analyze these differences in relation to geographic origin. Retrospective observational study of all primary care visits made in 26 urban health centers. Main variable: total number of health centre visits/year. Dependent variables: type of clinician requested; type of attention, and origin of immigrants. The independent variable was nationality. Statistics were obtained from the electronic medical records. The 4,933,521 appointments made in 2007 were analyzed for a reference population of 594,145 people (11.15 % immigrants). The adjusted annual frequency for nationals was 8.3, versus whereas 4.6 for immigrants. The immigrant population makes less use of primary care services than national population. This is evident for all age groups and regardless of the immigrants’ countries of origin. This result is important when planning health care resources for immigrant population.

## Introduction

The right to health services in equal conditions is a priority in the developed world and particularly in the countries of the European Union, of which Spain is a member state. Access to health services should be regardless of wealth, education, age or ethnic group, among other social markers. Yet, this is often not the case, at least as far as immigrant populations are concerned [1–3]. Spain has attracted a large number of economic migrants in recent years. As of 1 January 2010, immigrants in Spain accounted for 12.15 % of the total population and 12.79 % of that of the Autonomous Region of Aragon [4].

To date, the use of health services by immigrant populations has been studied by means of surveys or samples [3, 5–9]. These studies serve an important purpose but have clear methodological limitations, such as biases in sample selection, low response rates and different forms of biases in responses. These limitations may be overcome if health system utilization is studied by accessing national health insurance scheme databases and those containing electronic medical records available in primary care. This may even allow us to discover the part of the population that has not made use of health services in a given period. Three recent reviews have pointed out the need for this type of studies [5, 6, 10]. This approach is quite important because it will give us real evidence of possible inequities in access to health services and health outcomes in the immigrant population, particularly when legal access is acknowledged and encouraged by Spanish law. Additionally, the same law reminds public administrations that they are to direct their actions towards preventing discrimination against any collective that may have particular difficulty in accessing the Spanish National Health System services for cultural, linguistic, religious or social reasons.

The Spanish National Health System has two important features that make it particularly relevant for this type of study. It is a universal and practically free system (there is only a reduced co-payment for outpatient prescription drugs). The health system operates in the same way for the immigrant population, including those whose status in the country is irregular [11], which should improve real access to it [12, 13]. There is only one condition: to be registered on the census—this does not involve any risks to individuals who are in an irregular administrative situation. Universal health care is provided to all immigrants under the age of 18, to pregnant women and in any emergency situation.

The present study aims to overcome the previously-mentioned methodological difficulties, and has the purpose of gaining insight into the real use of primary care services made by both Spanish nationals and immigrants.

This will be very useful for health policy on immigration.

Its aims are:

1. to analyse the use of primary care services by immigrants compared to Spanish nationals, adjusted by age and sex;
2. to analyse the differences in frequency of visits to primary care in relation to geographic origin.

## Methods

This is a retrospective observational study of all primary care visits made during 2007 to 26 health centres of a large Spanish city, Zaragoza, with a population of 700,000 inhabitants.

The main variable is defined as the total number of health centre visits/year. The dependent variables were type of clinician with whom an appointment was requested (general practitioner, paediatrician, midwife, physiotherapist, dentist, social worker or for diagnostic tests); type of care requested: on demand (requested by patient), scheduled (by clinician), urgent (requested by patient), scheduled house call and urgent house call; and origin of immigrants. The independent variable was nationality. For the purpose of this study, an immigrant was defined as a person whose nationality was not Spanish (foreign national), regardless of his/her economic situation [14]. We grouped nationalities together for the purpose of analysis.

Statistics on patients with health care entitlements were obtained from the electronic medical records register (OMI©: Computerized Medical Office). Information on the population assigned to the studied health centres—regardless of their use of primary care services during 2007—was extracted from the central medical database of the regional health service.

Patient anonymity was guaranteed at all times. In order to match data from both registers, the Aragonese Identification Code was used to identify patients univocally.

Frequency rates of primary care visits were calculated adjusting for age and sex both for the immigrant and Spanish national populations. Rates were also standardized by the direct method, taking the Spanish population as a benchmark, according to data from the Spanish National Institute of Statistics (www.ine.es) on 1 January 2008, in order to prevent differences caused by population distribution. Statistical analysis tests were not required given that data covered the whole population.

This research has been approved by Ethic Committee of Clinic Research of Aragon.

## Results

The 4,933,521 appointments made in 2007 were analysed for a reference population of 594,145 individuals, of which 66,264 were immigrants (11.15 %). 547,524 were paediatric appointments (0–15 years) for a population of 71,114 children (10.87 % were immigrants).

The distribution of the studied population is given in Table 1.

**Table 1:** Distribution of the studied population by sex and origin

	Males	%	Females	%	Total	%
Spain	251,921	87.93	275,960	89.70	527,881	88.85
Foreign nationals	34,580	12.07	31,684	10.30	66,264	11.15
Latin America	10,775	3.76	14,196	4.61	24,971	4.20
Eastern Europe	10,072	3.52	10,309	3.35	20,381	3.43
Sub-Saharan Africa	5,907	2.06	2,431	0.79	8,338	1.40
North Africa	4,709	1.64	2,282	0.74	6,991	1.18
EU and USA/Canada	1,687	0.59	1,196	0.39	2,883	0.49
Asia/Oceania	1,430	0.50	1,270	0.41	2,700	0.45

Table 2 shows the adjusted frequency of visits (No. visits/100 persons/year) to health centres by type of clinician and care. The adjusted annual frequency of visits for the nationals was 833, whereas this was 458 for immigrants. The Asia/Oceania population had the lowest frequency (308), followed by the Eastern European (329), Sub-Saharan African (479), North African (479), the European Union (pre-2007)-USA/Canada (516) and Latin American (518) populations.

**Table 2:** Number of visits per 100 persons/year to health centres by type of clinician and attention type

	Spanish nationals	Immigrants	Sub-Saharan African	Asia/Oceania	Eastern European	Latin American	North African	European Union (pre-2007)-USA/Canada
Population	527,881	66,264	8,338	2,700	20,381	24,971	6,991	2,883
*Clinician*								
Primary Care Team appointments	833	458	479	308	329	518	479	516
GP^a^ appointments	506	305	324	207	203	359	327	323
Paediatric appointments	631	408	414	279	390	431	444	348
Nurse appointments	227	83	89	47	58	87	81	117
Midwife appointments	18	14	9	9	18	19	13	14
Physiotherapy appointments	2	0	0	0	0	1	1	1
Dental appointments	5	6	6	5	6	5	8	3
Social worker appointments	3	2	3	0	1	2	2	3
Diagnostic tests	65	41	41	34	33	43	37	59
*Attention type*								
On demand	529	306	317	212	222	353	311	332
Scheduled	213	105	108	68	73	117	101	123
Urgent	66	42	51	27	29	44	64	42
Scheduled house call	6	1	1	0	0	0	0	4
Urgent house call	19	5	3	1	4	3	2	14

^a^General practitioner (GP)

**Fig. 1:**
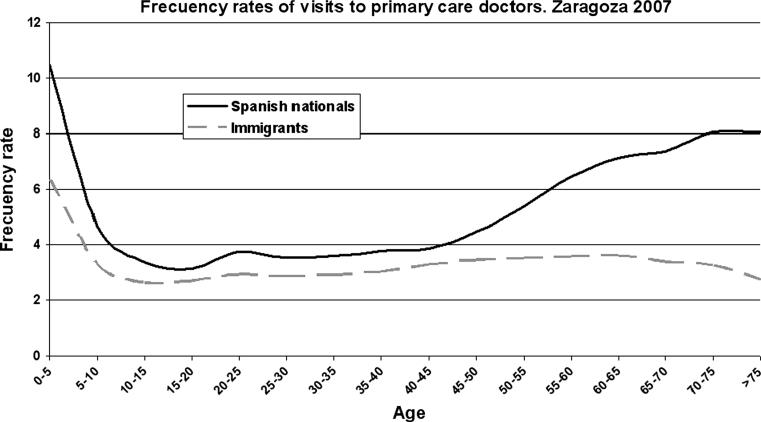
Frequency rates of visits to doctors by age

Figure 1 shows the frequency rate of visits to doctors by age.

**Fig. 2:**
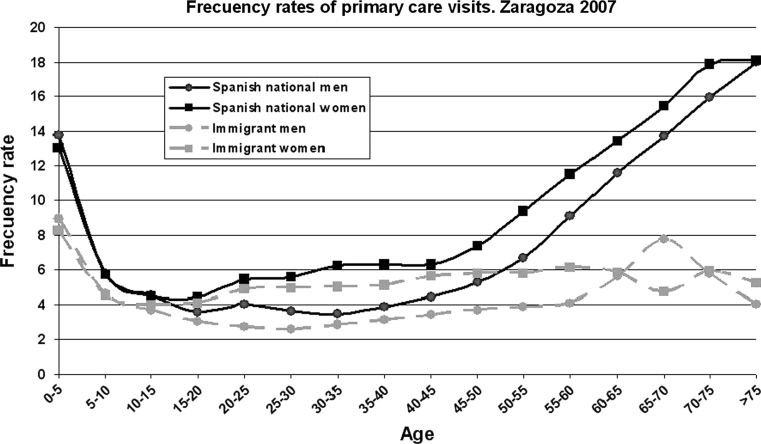
Frequency rate of primary care visits by age and sex

The rate with which women visit doctors is higher than for men in both national and immigrant populations. Figure 2 shows the same values in relation to age in total visits to Primary Care Teams.

## Discussion

The results of this study are conclusive: the immigrant population makes less use of primary care services than the population of Spanish nationals. This is evident for all age groups and regardless of immigrants’ countries of origin.

The city of Zaragoza is the capital of the Autonomous Region of Aragon, which, as all Spanish autonomous regions, is responsible for health care provision to its population. In recent years, Aragon has become home for a large number of immigrants who now have access to the health system in the conditions established by the law.

Our results are in agreement with other similar, recently-published studies focused on hospital utilization [15–17], prescription drug utilization [18, 19] and the utilization of primary care services [20] in Spain. In the systematic review by Uiters et al. [6], lower frequencies of primary care use were also found in the immigrant population, but with significant variance in the studies it covers.

This evidence contrasts with the general opinion that immigrant patients ‘overuse’ health services. Esteva [21] surveyed a sample of 159 Spanish General Practitioners, of whom 81 % agreed that “immigrants visit hospital accident and emergency departments and primary care practices with greater frequency than other registered patients”. This perception does not agree with the data obtained in our study. These stereotypes, which also exist in other countries such as the US, have recently been refuted by other authors [22].

However, despite coinciding findings, we have also observed a number of differences with regard to other studies. Other authors using surveys have found that the use of primary care services by immigrants is only slightly lower than the use made by Spanish nationals [8, 9, 23, 24] or even higher [5]. It is interesting to observe that when working with population data, the use of services by immigrants is found to be much lower. However, these differences tend to disappear or are inverted when the data is gathered from survey-based studies. This may be due to possible biases in sample selection or in responses.

‘Underuse’ of health services by immigrants in our study can be explained by different factors.

First, there is an a priori unexpected difficulty to real access to the health system. Although Rivera [23] and Carrasco-Garrido [3, 25] defend the notion that there are no major barriers to access, other authors assert that there are barriers caused by cultural differences, language, legal status, lack of familiarity with health care provision services, employment situation and timetable incompatibility [26, 27] that hinder access. As a denominator, our study used the population recognized as holding the public health insurance card; therefore, a possible ‘legal’ barrier to access does not seem to be an explanation.

Another possible explanatory factor would be incompatibility with practice hours, which may be caused by lower levels of stable employment conditions, where it becomes more difficult for people in this situation to take time off work to go to the doctor. A qualitative analysis would be required to confirm this hypothesis in order to explore these aspects.

However, if we assume that the immigrant population has a lower socio-economic level than that of the Spanish population, it would seem that immigrants should have lower levels of health, and therefore greater needs for health care [9, 12, 28]. The low visit frequency rate in the immigrant population compared to Spanish nationals found in our study could respond to the classic inverse care law described by Hart [29]: “the availability of good medical care tends to vary inversely with the need of the population served”. Another possibility could be that, despite the social determinants to which immigrants are subjected, their health may be better that expected.

In other words, it could be thought that immigrants make fewer visits to the doctor because they are healthier. This contradicts the theory that associates lower socio-economic level with worse general health [30]. There are numerous studies pointing out that immigrants generally have a better level of health than host populations because the healthiest are more likely to migrate [31–37] (“healthy migration effect”) and this population group has better lifestyles [3, 23, 32].

The lower use of health services could be also based on the different concepts of and attitudes towards health and illness in both groups. This idea is supported by the different rates found according to region of origin and culture. For example, immigrants of Asian origin have much lower visit frequencies than Latin Americans. This could also be due to a greater use of traditional medicines by some of these groups.

Health and illness are socially-construed concepts, and therefore vary greatly according to the cultural environment. Immigrants may generally have a more ‘utilitarian’ concept of health, associated with the ability to work. Preventive activities, whether for children or adults, may not be seen to be priorities as they are not acknowledged as needs, at least in their first years in the host country. This would derive in lower rates of scheduled visits and fewer on-demand visits, particularly those monitoring the evolution of chronic processes. Following this premise, the health culture of the countries of origin should be considered in public health programs for prevention and care.

Health care in the immigrants’ countries of origin is generally characterized by major deficiencies, which make them relegate medical attention to processes they consider to be more serious, while treating “banal” conditions themselves with home remedies. This ‘habit’ may endure once these people arrive in Spain, particularly during the first years. With time, immigrants may adapt and assimilate Spain’s culture of greater health service utilization. Studies with individuals’ follow-up over time will be necessary to confirm this trend. Likewise, studies dealing with the children of immigrants or ‘second generations’ will be very useful.

Very different rates of visit frequency have been observed depending on the immigrants’ region of origin. Among the foreign national population, the groups with highest frequency rates are those from ‘rich countries’ (with a similar socio-economic level and concept of health-illness to the Spanish population) and Latin Americans (with significant cultural and linguisitc similarities). At the opposite end of the scale are Asian immigrants (with great linguistic and cultural differences) and the population from Eastern Europe (mainly Romanians, who learn Spanish very quickly). This variability opens up interesting research opportunities. Although included with Asians, immigrants from Oceania form a very small minority and their behaviour is not reflected in this discussion. For cultural reasons, this is more likely to resemble that of other Western nations.

The main methodological strength of this study is that it analyses real visits for the entire reference population (whether or not they have made use of the health system during the year) and measures the impact on the real utilization of the public health system by immigrants and Spanish nationals. Likewise, as the demographic information relative to patients is available, the statistics could be adjusted for age and sex. This methodology adds a totally objective and quantifiable perspective [20]. Studies based on surveys may have significant bias in sample selection or in the quality of the collected information [3, 6, 9, 23, 38].

Another strength is the very high volume of statistics taken from the studied population, both national and immigrant, making up for 5 million appointments at the region level. It is thus a useful tool for health planning of a country. This has enabled the immigrant population to be studied according to their origin. Although the number of immigrants from Latin America, Eastern Europe and North Africa form the bulk, information is available on all immigrant populations, regardless of their origin.

As limitations to the study, we would point out that bias could occur as the result of one health insurance card being used by several immigrants, who visit a health centre using a card belonging to a family member or acquaintance. This would cause an overestimation of the frequency of visits by immigrants compared to nationals, leading to a widening of the differences found. However, this phenomenon has been reduced after Spanish Law 4/2000 was passed governing the rights and freedoms of foreign nationals in Spain and their social integration. This has made their access to the public health system easier, given that the only requisite is that they should be registered on the census [11].

Another limitation is that we are unable to adjust the figures for morbidity, making it difficult to speak of the adaptation of either group in this regard. Further studies regarding this aspect are being developed at present.

In summary, in a universal, free health care system, immigrants show a lower frequency of visits than that of Spanish nationals for any age group, regardless of sex and geographic origin. There is an important variance depending on geographic background that could be studied in more detail. In this regard, cultural factors, varying access or the concepts of health and illness may play an important role. Such studies should be assessed when planning health policies dealing with access to health services.
